# Propensity to endoplasmic reticulum stress in deer mouse fibroblasts predicts skin inflammation and body weight gain

**DOI:** 10.1242/dmm.049113

**Published:** 2021-10-18

**Authors:** Youwen Zhang, Chang-uk Lim, Vitali Sikirzhytski, Asieh Naderi, Ioulia Chatzistamou, Hippokratis Kiaris

**Affiliations:** 1 Department of Drug Discovery and Biomedical Sciences, College of Pharmacy, University of South Carolina, Columbia, SC 29208, USA; 2 Department of Pathology, Microbiology and Immunology, School of Medicine, University of South Carolina, Columbia, SC 29209, USA; 3 Peromyscus Genetic Stock Center, University of South Carolina, Columbia, SC 29208, USA

**Keywords:** Endoplasmic reticulum stress, Unfolded protein response, High-fat diet, Inflammation, Metabolic disorders

## Abstract

The unfolded protein response (UPR) is involved in the pathogenesis of metabolic disorders, yet whether variations in the UPR among individuals influence the propensity for metabolic disease remains unexplored. Using outbred deer mice as a model, we show that the intensity of UPR in fibroblasts isolated early in life predicts the extent of body weight gain after high-fat diet (HFD) administration. Contrary to those with intense UPR, animals with moderate UPR in fibroblasts and therefore displaying compromised stress resolution did not gain body weight but developed inflammation, especially in the skin, after HFD administration. Fibroblasts emerged as potent modifiers of this differential responsiveness to HFD, as indicated by the comparison of the UPR profiles of fibroblasts responding to fatty acids *in vitro*, by correlation analyses between UPR and proinflammatory cytokine-associated transcriptomes, and by BiP (also known as HSPA5) immunolocalization in skin lesions from animals receiving HFD. These results suggest that the UPR operates as a modifier of an individual's propensity for body weight gain in a manner that, at least in part, involves the regulation of an inflammatory response by skin fibroblasts.

This article has an associated First Person interview with the first author of the paper.

## Abstract (precis)

**Highlighted Article:** Using outbred deer mice, we show that the unfolded protein response operates as a modifier of propensity for body weight gain in a manner involving the regulation of an inflammatory response by skin fibroblasts.

## INTRODUCTION

The regulation of body weight gain is an extremely complex process that involves the neurochemical and hormonal regulation of appetite, the absorption of nutrients and calorie-rich compounds, and their processing, distribution and, ultimately, storage or utilization in the periphery (Lund et al., 2020; Berthoud et al., 2020). Extreme body weight gain, owing to a combination of environmental, dietary and genetic factors, results in obesity, which is associated with increased adiposity and, ultimately, the development of metabolic pathologies, such as hepatic steatosis, insulin resistance and diabetes, and cardiovascular disease. Intrinsic to the pathogenesis of obesity is the development of meta-inflammation, which involves the moderate chronic inflammation following increased adiposity and contributes to insulin resistance and metabolic disease (Kawai et al., 2021; Kojta et al., 2020; Ye et al., 2012).

Central to the onset and progression of metabolic disease is the unfolded protein response (UPR), which constitutes a conserved homeostatic response developing in cells to resolve endoplasmic reticulum (ER) stress (Walter and Ron, 2011). Numerous studies in mouse models involving the induction of qualitative changes, such as the loss or gain of function of specific UPR-related genes, show that ER stress is implicated in several metabolic pathologies. To that end, loss of activity by gene deletion or overexpression of dominant-negative gene versions was sufficient to induce metabolic phenotypes such as hepatic steatosis or diabetes in mouse models (Almanza et al., 2019; Zhou and Liu, 2014; Rutkowski et al., 2008). Moreover, the UPR is associated with the regulation of the inflammatory response, which in turn modulates metabolic disease (Amen et al., 2019; Smith, 2018; Barabutis, 2020; Zhang et al., 2021). Yet, how subtle variations in the UPR occurring naturally among individuals influence the propensity for the development of metabolic pathologies remains unexplored. In the present study, we evaluated whether inherent variations in the UPR among genetically diverse individuals impact the propensity to body weight gain and the induction of inflammation following administration of a high-fat diet (HFD). To address this, we utilized outbred deer mice (*Peromyscus maniculatus*) that exhibit highly variable, yet tightly coordinated, UPR among individuals (Havighorst et al., 2019; Zhang et al., 2019). Animals were assigned to different groups according to the UPR profiles in their fibroblasts, and then the response to the HFD was evaluated by determining body weight gain and the induction of systemic inflammation. Whether the intensity of the UPR correlates with the efficiency of ER stress resolution was also evaluated, and whether this predicts the strength of the proinflammatory response was assessed. Our results collectively integrated UPR profile to body weight gain by identifying skin fibroblasts as essential modulators of the response to the HFD through differential induction of inflammation.

## RESULTS

### UPR variation and ER stress resolution in fibroblasts

**Fig. 1.**
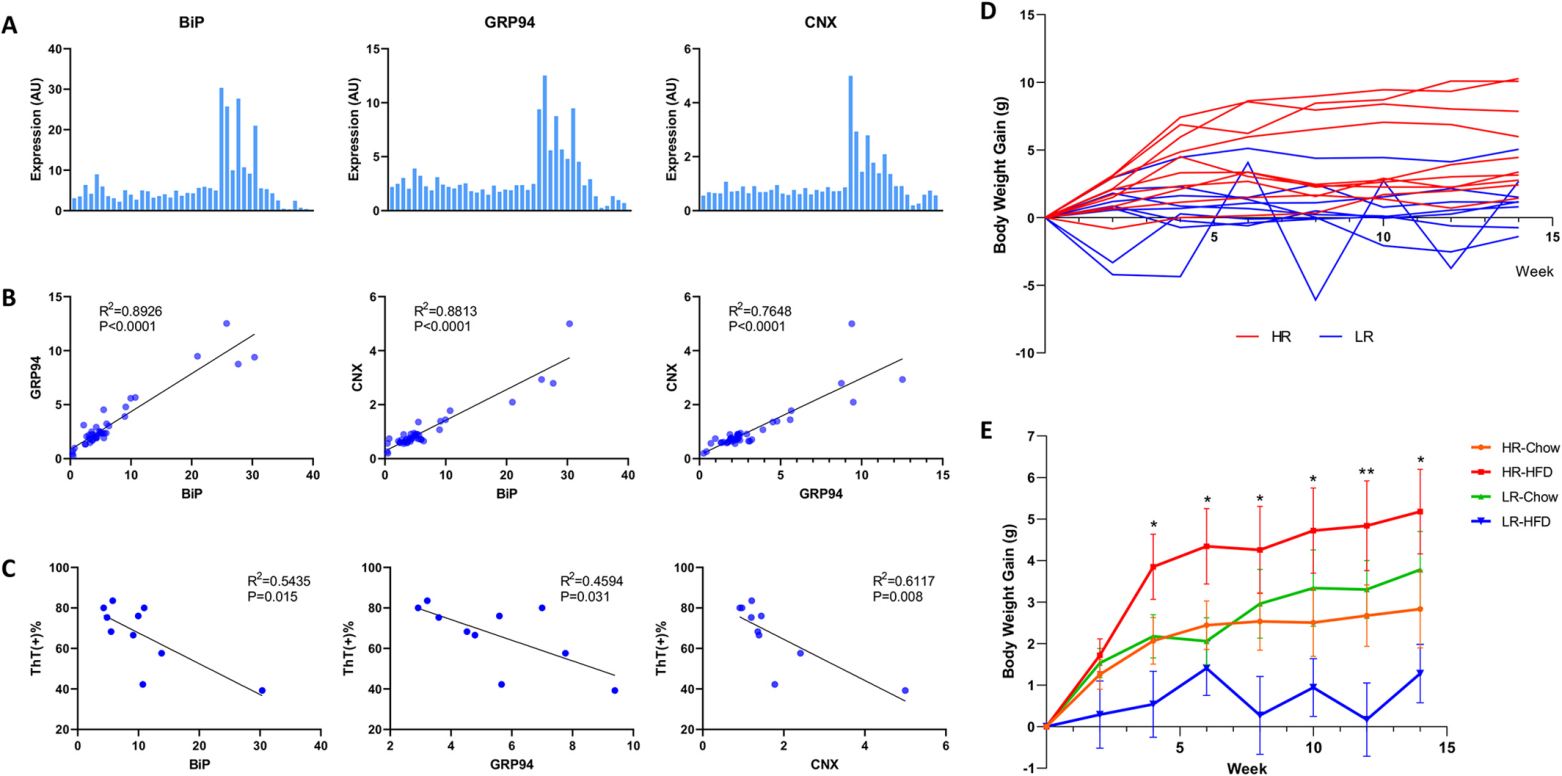
**Variations in the unfolded protein response (UPR) and body weight gain in deer mice.** (A) Expression of BiP, GRP94 and calnexin (CNX; also known as CANX) in primary fibroblasts isolated at puberty from *P. maniculatus* after exposure to tunicamycin (5 μg/ml) for 5 h. All expression values [arbitrary units (AU)] were normalized in relation to GAPDH (*n*=42; 33 males and nine females). Each bar represents an individual animal. (B) Coordinated expression of UPR-associated genes in primary fibroblasts of *P. maniculatus* (*n*=42; 33 males and nine females). Pairwise comparisons in expression of endoplasmic reticulum (ER)-stress-related genes after exposure of cells to tunicamycin. *R*^2^ values from Pearson's correlation and *P*-values are shown. (C) Correlation between ratios of Thioflavin T (ThT)-positive primary fibroblasts of *P. maniculatus* after exposure to ThT (4 µM) and tunicamycin, and expression of ER chaperones after exposure to tunicamycin (*n*=10, males). *R*^2^ values from Pearson's correlation and *P*-values are shown. (D) Body weight gain in genetically diverse *P. maniculatus* after high-fat diet (HFD) consumption for 14 weeks [*n*=10 in high UPR responders (HR), *n*=9 in low UPR responders (LR), all males]. Highly variable response was recorded and most of the HR gained more body weight compared to the LR. (E) Comparison of body weight gain among four different groups: HR-Chow (*n*=7, males), high responders fed a regular chow diet; HR-HFD (*n*=10, males), high responders fed a high-fat diet; LR-Chow (*n*=7, males), low responders fed a regular chow diet; LR-HFD (*n*=9, males), low responders fed a high-fat diet. *P<0.05, **P<0.01. *P*-values were calculated with ordinary one-way ANOVA and Tukey's multiple comparisons test.

To record the UPR profile, primary fibroblasts were isolated from *P. maniculatus* at weaning, exposed to tunicamycin, which induces canonical UPR, and the levels of the chaperones BiP (also known as HSPA5), GRP94 (also known as HSP90B1) and calnexin were evaluated. As expected, highly variable UPR among different individuals was detected that followed a positive linear correlation in the pairwise comparisons of all chaperones. This in turn identified some animals as high (HR) and others as low (LR) UPR responders (Fig. 1A,B).

Initially, we sought to explore the implications of intense versus moderate UPR at the cellular level, in terms of stress resolution. It is plausible that intense UPR may indicate a state in which intense ER stress has been induced due to inappropriate protein folding. Alternatively, it may reflect the enhanced ability for more efficient resolution of ER stress due to adequate stimulation of the cellular protein quality control mechanisms (Reich et al., 2020; Lin et al., 2008; Chen et al., 2017; Walter and Ron, 2011). It is plausible that such questions could be addressed by subjecting a diverse set of specimens displaying variable UPR to the same misfolding protein-inducing stimulus and then by correlating chaperone expression with the abundance of unfolded proteins. Thus, we exposed cells to Thioflavin T (ThT), a fluorescent chemical compound that shows enhanced fluorescence signals when it binds to protein aggregates (Beriault and Werstuck, 2013), and measured ThT signals in cells by flow cytometry. We hypothesized that, by evaluating whether and how ThT fluorescence correlates with the chaperone expression, we could directly assess the implications of UPR intensity in ER stress resolution. As shown in Fig. 1C, a significant negative correlation was revealed between the levels of each of BiP, GRP94 and calnexin, and ThT abundance, suggesting a more efficient ER stress resolution when chaperone levels are high.

### UPR in fibroblasts predicts body weight gain in HFD-fed deer mice

Having established that differential UPR is associated with the efficiency by which ER stress is resolved, at least in primary fibroblasts, we then tested whether this bears implications at the organismal level and especially on body weight gain. Therefore, cohorts of animals were established in which the UPR had been characterized in the fibroblasts at weaning. Subsequently, animals received a HFD or a regular chow diet, and body weight gain and food consumption were monitored. HFD is a known activator of the UPR and an established modifier of the onset of metabolic pathologies (Matveyenko et al., 2009; Li et al., 2012; Usui et al., 2012). Two experimental groups were defined per diet and included the individuals in which the levels of chaperones BiP, GRP94 and calnexin in the fibroblasts were in the higher 50th percentile (HR) or those that belonged to the lower 50th percentile (LR). As shown in Fig. 1D,E, contrary to laboratory mice (*Mus musculus*), which, depending on the specific strain, may show an increase in body weight after HFD consumption that can exceed 50% (Kahle et al., 2013; Rossmeisl et al., 2003; Montgomery et al., 2013), deer mice gained body weight only moderately, albeit variably, which indicates a rather robust regulation of metabolism and retention of homeostasis, which restricts severe obesity. Thus, in this model, it is plausible to speculate that the impact of chronic inflammation (or meta-inflammation) as a confounding factor in disease development is reduced and can be dissociated because body weight gain was overall minimal.

When body weight gain data were analyzed with respect to the UPR responsiveness of fibroblasts, it was revealed that the HR group gained significantly more body weight than the LR group (Fig. 1D,E), and this body weight gain paralleled food consumption (Fig. S1).

### Low UPR in fibroblasts is associated with higher levels of proinflammatory markers in the animal sera

**Fig. 2.**
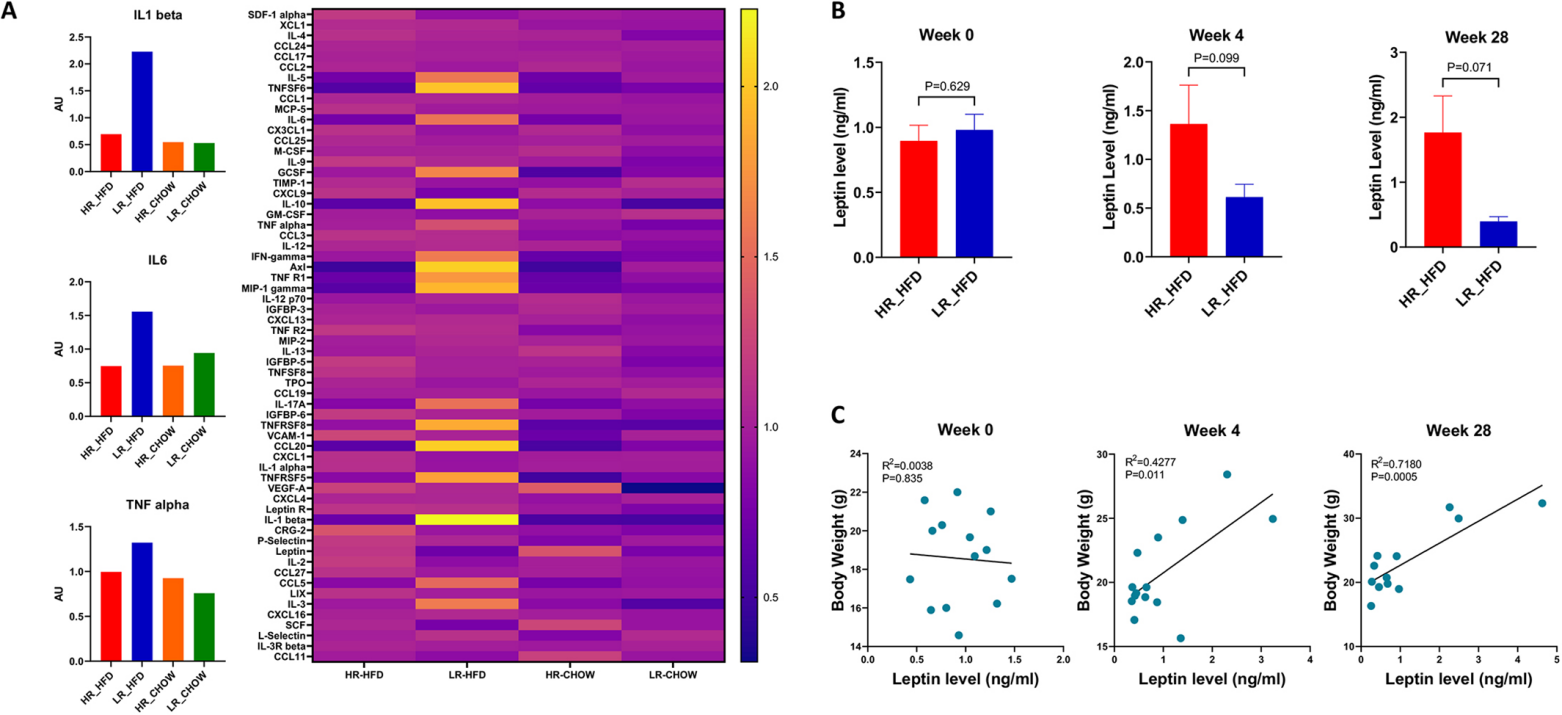
**Proinflammatory cytokines in the sera of deer mice.** (A) Heat map representing color-coded relative levels of 62 cytokines in the sera of four different groups: HR-Chow (*n*=6, four males and two females); HR-HFD (*n*=7, five males and two females); LR-Chow (*n*=6, four males and two females); LR-HFD (*n*=7, four males and three females). The sera were pooled together for each group for the cytokine-array measurements. The left column shows the relative levels of three proinflammatory cytokines, IL1 beta, IL6 and TNF alpha, in sera of each group. (B) Leptin levels in sera of HR (*n*=7, five males and two females) and LR (*n*=7, four males and three females) before and after 4 weeks and 28 weeks of HFD consumption detected with a leptin ELISA kit. *P*-values were calculated with unpaired Student's *t*-test. (C) Correlation between serum leptin level and body weight in HR (*n*=7, five males and two females) and LR (*n*=7, four males and three females) before and after 4 weeks and 28 weeks of HFD consumption. *R*^2^ values from Pearson's correlation and *P*-values are shown.

The fact that inflammation is inherently linked to the consumption of the HFD, obesity and appetite led us to explore whether and to which extent distinct proinflammatory responses differentiate LR and HR animals following HFD consumption. To that end, we utilized a mouse cytokine array to simultaneously assess the levels of several proinflammatory cytokines in the animal sera. Surprisingly, we unveiled a clear trend, applicable to almost all cytokines evaluated, for induction, specifically in the group of LR animals and only after the administration of the HFD (Fig. 2A). Thus, HR animals tolerate the HFD and respond by gaining body weight, whereas LR animals develop an inflammatory response instead, which prevents them from gaining body weight. Noteworthy, serum lipids, such as total and free cholesterol, as well as high-density lipoprotein (HDL) and low-density lipoprotein (LDL)/very-low-density lipoprotein (VLDL) cholesterol, were also evaluated, but despite a trend for higher levels in the LR animals, the differences were insignificant (Fig. S2).

### Leptin levels do not predict body weight gain in HR and LR animals

It is known that, depending on the context and the severity of the response, inflammation can produce contrasting effects in appetite and obesity. Inflammation is associated with anorexia and appetite loss (Gautron and Layé, 2010; Braun and Marks, 2010), yet chronic inflammation has been linked to the development of obesity by mechanisms involving leptin resistance and appetite increase (De Souza et al., 2005; Thaler et al., 2013; Kleinridders et al., 2009). In this deer mouse model, leptin regulation operated normally because its levels were similar in both groups at the beginning of the study, but, after 4 weeks on the HFD, leptin levels were higher in the HR animals that had gained more body weight (Fig. 2B). Furthermore, the levels of circulating leptin were proportional to body weight (Fig. 2C). These observations support the notion that the gain of body weight in deer mice in response to the HFD is not modulated by the onset of obesity, dissociating the secondary consequences of increased adiposity and chronic inflammation from the acute induction of inflammation in response to the HFD. Furthermore, they point to the operation of a regulatory network contributing resistance to a subset of animals, specifically the LR animals, which do not tolerate the HFD well, develop an inflammatory response and do not gain body weight.

### Induction of inflammation in the skin of deer mice by the HFD

**Fig. 3.**
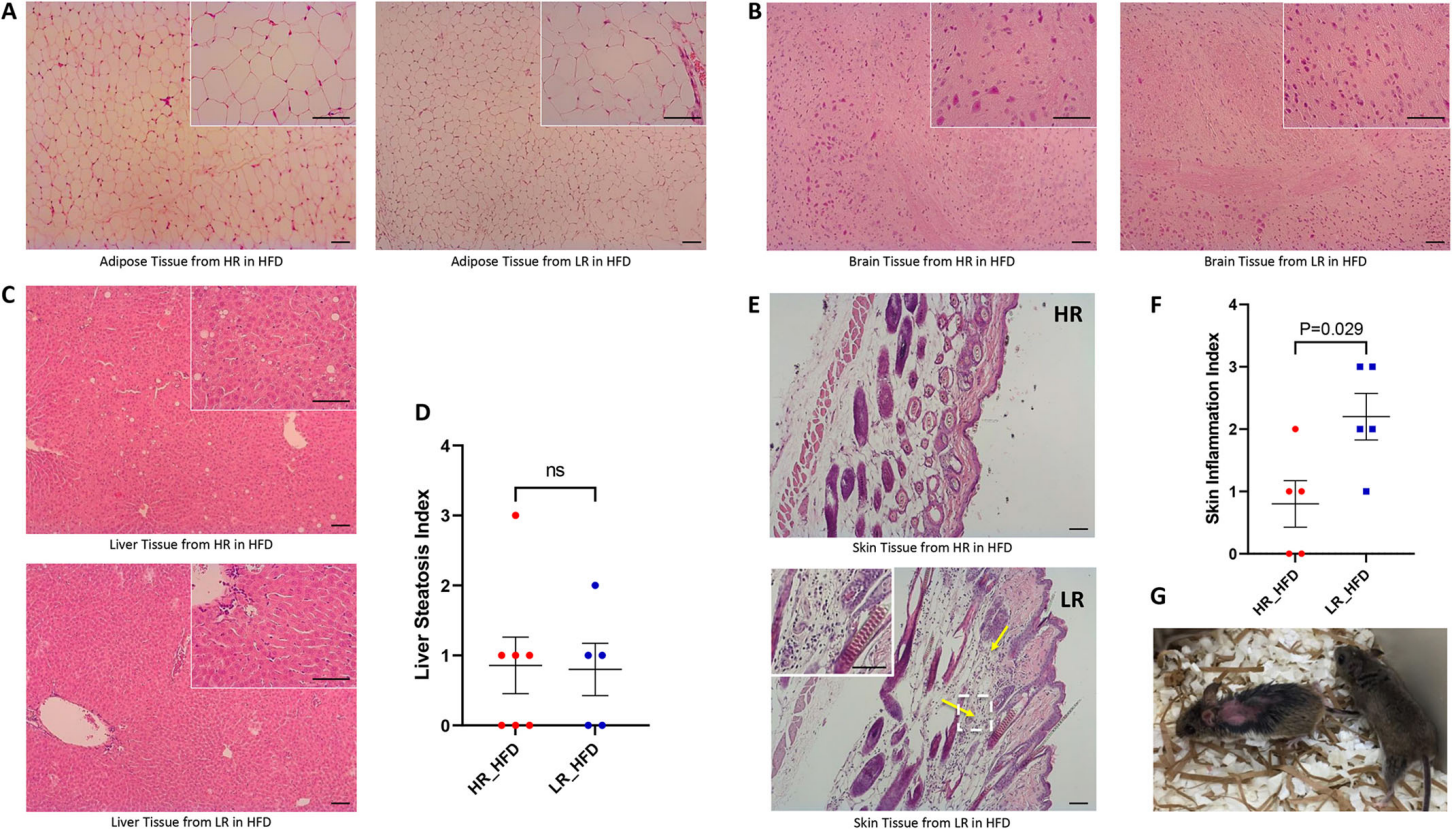
**Histology of liver, brain, adipose and skin tissue in deer mice after HFD administration.** (A) H&E-stained sections of subcutaneous fat from HR (*n*=7, five males and two females) and LR (*n*=7, four males and three females; among them, two males died after 8 weeks and 18 weeks of HDF administration) after 28 weeks of HFD consumption. No evidence of inflammation was seen in both groups. (B) H&E-stained sections of brain tissue from HR (*n*=7, five males and two females) and LR (*n*=5, two males and three females) after 28 weeks of HFD consumption. No evidence of inflammation was seen in both groups. (C) H&E-stained sections of livers from HR (*n*=7, five males and two females) and LR (*n*=5, two males and three females) after 28 weeks of HFD consumption. No evidence of inflammation was seen in both groups. (D) Liver steatosis index in HR (*n*=7, five males and two females) and LR (*n*=5, two males and three females) after 28 weeks of HFD consumption. ns, no significant difference shown by unpaired Student's *t*-test. (E) H&E-stained sections of skin tissue from HR (*n*=5, males) and LR (*n*=5, males) after 14 weeks of HFD consumption. Minimal inflammation was observed in the skin section of HR (top). A high degree of inflammation was observed in the skin section of LR (bottom). Yellow arrows indicate high immune cell-accumulating areas; the area in the dashed line box is also shown in the upper-left corner at a higher magnification. Scale bars: 50 μm. (F) Skin inflammation index in HR (*n*=5, males) and LR (*n*=5, males) after 14 weeks of HFD consumption. The *P*-value was calculated by unpaired Student's *t*-test. (G) Some of the LR developed skin lesions after HFD consumption. No skin lesions were seen in the HR after HFD consumption.

The observation that the UPR response of fibroblasts early in life reliably predicts the propensity for body weight gain in HFD-fed deer mice is consistent with two plausible mechanisms. First, it is conceivable that cultured fibroblasts, after tunicamycin treatment *in vitro*, recapitulate the response of certain peripheral organs to the HFD, operating as surrogates. To that end, the induction of inflammation was assessed histologically in brain, liver and adipose tissue, which represent established target tissues of the HFD in mice. As shown in Fig. 3A-C, evidence of inflammation was not recorded in any of these tissues, implying that the intensity of the proinflammatory response does not represent a likely mechanism that could explain the differential effects of the HFD in the deer mice. Furthermore, the expression of various UPR markers was also evaluated in these tissues after HFD consumption, but no association with the UPR in fibroblasts was identified (Fig. S3). Hepatic steatosis did develop occasionally, in ∼30% of the animals, but was not associated with body weight gain, indicating that liver dysfunction proceeds independently of the body weight gain in this model (Fig. 3D). Whether the lower incidence of steatosis in this study compared to our earlier analysis (Havighorst et al., 2019) is due to the genetic diversity of the animals or due to the fact that animals in which the UPR was not coordinated were purposely selected, introducing a confounding factor in steatosis, remains unclear. Nevertheless, a clear association between body weight gain and UPR classification of fibroblasts was unveiled in a manner according to which intense UPR in fibroblasts early in life predicts a more pronounced gain of body weight.

According to an alternative possibility, the fibroblasts, instead of operating as surrogates, are contributing directly as modifiers of body weight gain via mechanisms implicating the differential UPR-associated inflammation. Various considerations are supportive of this notion. First, the fibroblasts are the most abundant cell type in the body and, thus, even moderate changes cumulatively may be sufficient in inflicting biologically relevant responses. Skin, from which fibroblasts in this study were isolated, is particularly rich in fibroblasts, and skin fibroblasts were shown to respond differently in different diets in mice, such as a ketogenic diet or a HFD (Salzer et al., 2018). In addition, the induction of inflammatory response in the skin by special diets has been established, identifying this organ as responsive to dietary manipulations (Herbert et al., 2018; Rosa et al., 2018). Finally, in people, an extreme and rather rare severe inflammatory reaction in the skin that has been associated with ketogenic diets has also been described in some individuals and designated as prurigo pigmentosa (Beutler et al., 2015; Alshaya et al., 2019). In line with this evidence, in deer mice, we occasionally noted macroscopically skin lesions in the animals receiving the HFD that had been characterized as LR (Fig. 3G).

**Fig. 4.**
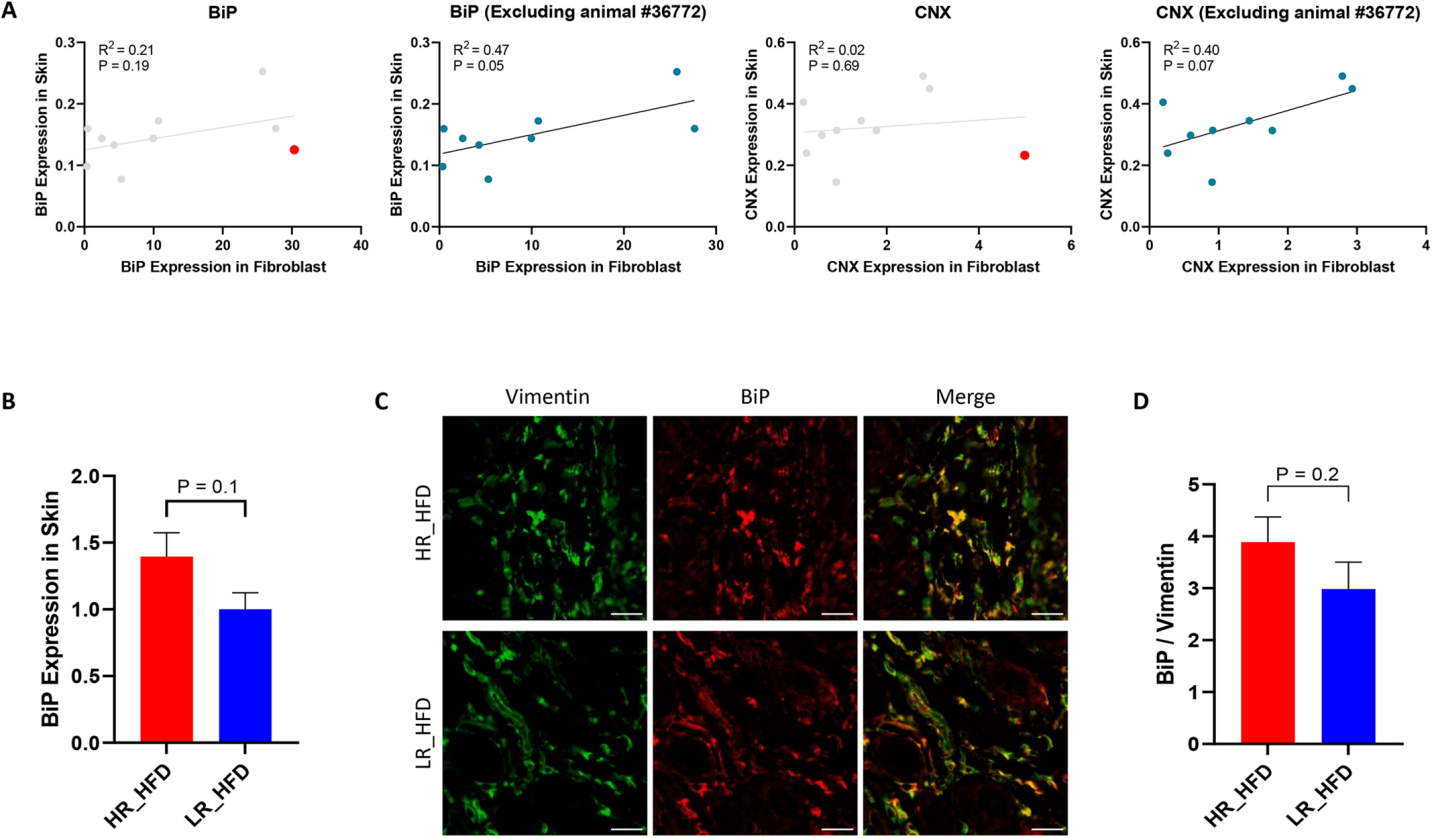
**UPR in the skin of deer mice after HFD administration.** (A) Correlation of ER chaperone (BiP and CNX) expression between skin tissue and primary fibroblasts of deer mice (*n*=10, males) after HFD consumption measured by RT-qPCR. Animal #36772 (red dot) was an apparent outlier and was either included (left) or excluded (right) in the analyses. *R*^2^ values from Pearson's correlation and *P*-values are shown. (B) BiP expression in skin tissue of HR (*n*=5, males) and LR (*n*=5, males) after HFD consumption measured by RT-qPCR. The *P*-value was calculated by unpaired Student's *t*-test. (C) Colocalization of BiP protein (red) with the fibroblast marker vimentin (green) in the skin tissue of HR (*n*=5, males) and LR (*n*=5, males) after HFD consumption. Scale bars: 25 μm. (D) Comparison of BiP colocalization with vimentin in the skin tissue of HR (*n*=5, males) and LR (*n*=5, males) after HFD consumption. The *P*-value was calculated by unpaired Student's *t*-test.

To explore further the operation of this mechanism, we analyzed histologically skin biopsies of animals that received the HFD for evidence of inflammation. Evidence of macrophage infiltration was revealed in almost all animals that had received the HFD, which was more prominent in the LR group (Fig. 3E,F). Assessment of BiP expression in fibroblasts by immunofluorescence was more prominent, albeit insignificantly, in the skin of the HR compared to the LR animals and also exhibited a trend for positive correlation with BiP expression of fibroblasts early in life following tunicamycin treatment *in vitro* (Fig. 4). Thus, we conclude that skin fibroblasts represent good candidates for operating as modifiers of the UPR in response to a HFD.

### Fibroblasts respond differently to fatty acids *in vitro* in relation to their UPR profile

**Fig. 5.**
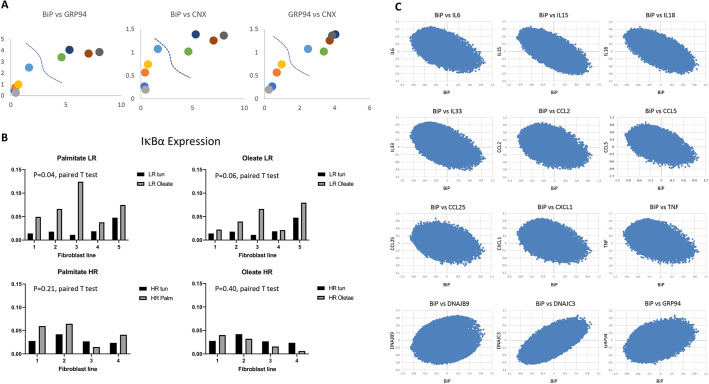
**UPR and inflammatory response in cultured fibroblasts.** (A) Correlation between the expression of ER chaperones (BiP, GRP94 and CNX) in each primary fibroblast line from an individual animal after tunicamycin treatment (*n*=9, five males and four females). Dot colors indicate animal identity. The HR are on the right side of the dashed lines and the LR are on the left. (B) IκBα expression in the primary fibroblast lines (*n*=9, five males and four females) treated with palmitate/oleate or tunicamycin measured with RT-qPCR. Black bars show IκBα expression induced by tunicamycin in each fibroblast line; gray bars show IκBα levels induced by palmitate/oleate. *P*-values were calculated with paired Student's *t*-test. (C) Whole-transcriptome coordination between *Il6*, *Il15*, *Il18*, *Il33*, *Ccl2*, *Ccl5*, *Ccl25*, *Cxcl1*, *Tnf*, *Dnajb9*, *Dnajc3*, *GRP94* and *BiP*. Scatterplots showing the *R* (Pearson's) for the transcripts correlated with each gene of the whole transcriptome between the designated comparison (*n*=10, males).

To test whether fibroblasts induce a proinflammatory response following fatty acid administration *in vitro* and whether this response is implemented differentially between the LR and HR fibroblasts, we exposed fibroblasts that were characterized in terms of their UPR profile to the fatty acids oleate and palmitate and monitored the levels of IκBα (also known as NFKBIA) (Fig. 5A). As shown in Fig. 5B, a more prominent induction of IκBα, a reporter of NFκB activity that mediates the inflammatory response, was recorded in the LR compared to the HR fibroblasts. This effect was recorded with palmitate (*P*<0.05) and marginally with oleate (*P*=0.06). These observations imply the existence of an inverse relationship between UPR gene expression and the levels of proinflammatory cytokines in primary, unchallenged fibroblasts from genetically diverse animals.

### BiP expression negatively correlates at the whole transcriptome with various proinflammatory cytokines

To test these notions further, *P. maniculatus* fibroblasts exhibiting a diverse UPR response were analyzed at the whole-transcriptome level for the mode of expression of the major chaperone BiP and several proinflammatory cytokines. Pairwise scatterplots were then developed, depicting the correlation of each transcript in the transcriptome between BiP and a roster of proinflammatory cytokines (Zhang et al., 2019). As shown in Fig. 5C, a negative correlation between the BiP-associated transcriptome and each of the transcriptomes associated with IL6, IL15, IL18, IL33, CCL2, CCL5, CCL25, CXCL1 and TNF in cultured skin fibroblasts was readily unveiled, suggesting that a more prominent induction of UPR is linked to a modest inflammatory response. However, for DNAJB9, DNAJC3 and GRP94, which are components of canonical UPR, a positive correlation with BiP was maintained, which is consistent with the notion that not only the expression between these chaperones is correlated in outbred species but also that the whole transcriptome coordinated with them maintains the same mode of expression (Fig. 5C).

## DISCUSSION

**Fig. 6.**
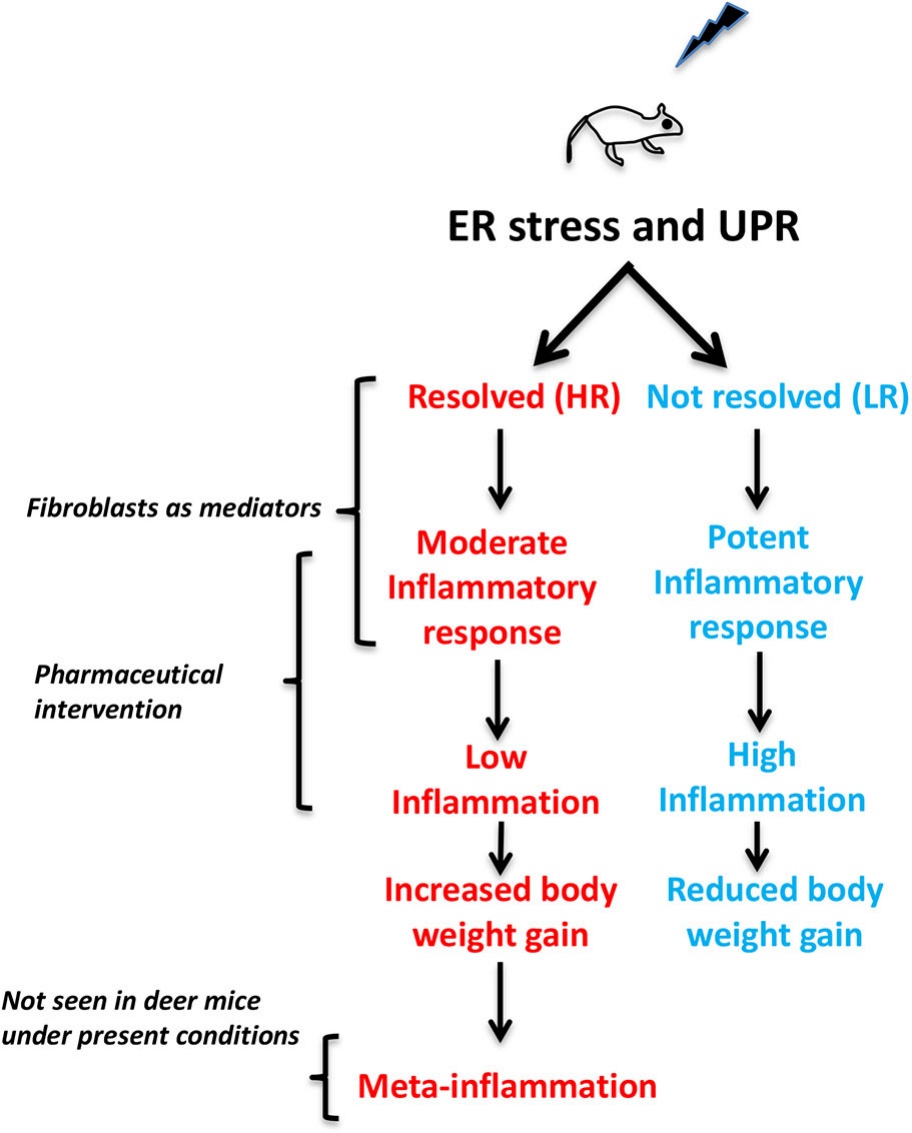
**ER stress and UPR modulate inflammatory response and body weight gain.** Stress inducers such as HFD cause ER stress and UPR in deer mice. The HR with higher ER chaperone expression have higher ability to resolve ER stress. They have moderate inflammatory response, low inflammation and increased body weight gain, which may induce meta-inflammation at a later stage, but it was not seen in the deer mice under the conditions. In contrast, the LR have lower ability to resolve ER stress. They have potent inflammatory response, high inflammation and reduced body weight gain.

Despite the availability of unequivocal evidence linking ER stress to metabolic pathologies, how inherent variations in the UPR influence their onset remains poorly understood. Studies in genetically diverse species permit the assessment of how quantitative differences in the UPR influence the susceptibility to metabolic disease. By using outbred deer mice as a model, we provided evidence that skin, and probably other tissue-resident fibroblasts, operate as modifiers of the propensity for diet-induced body weight gain. Contrary to conventional laboratory mice, in which high-calorie diet is linked to meta-inflammation, this model dissociates the effects of chronic, adiposity-associated meta-inflammation from the inflammatory response triggered directly by dietary lipids because prolonged HFD administration was insufficient to cause histologically detectable inflammation in the liver, the brain or the adipose tissue. This discrepancy in the response to the HFD between deer mice and conventional mice resembles the response of human populations, in which high variation is recorded, which in turn restricts the translational value of the findings obtained from laboratory mice (Lai et al., 2014). Such limitation of inbred strains is also exemplified by the observation that in outbred Sprague Dawley rats, obesity following HFD was recorded only in half of the animals (Levin et al., 1997). Consistently with the present findings, the inherent variations in the UPR and the efficiency of ER stress resolution in fibroblasts participate in the regulation of body weight gain by a mechanism in which, when ER stress is not adequately resolved, the inflammatory response is more intense. Conversely, efficient resolution of ER stress is associated with better tolerance for high-calorie diet and, ultimately, increased body weight gain (Fig. 6).

Genomic variations in UPR-associated genes emerge as good candidates for causing the recorded variability in the intensity of the UPR. Nevertheless, how this translates to differential inflammation and that in turn is recorded, at least primarily, in skin fibroblasts remains obscure. Considering earlier observations suggesting that high-altitude deer mice exhibit more intense UPR (Havighorst et al., 2019), it is plausible that these findings possess evolutionary implications: in environmental conditions in which nutrient resources are sparse, the increased prevalence of individuals with more intense UPR may facilitate higher food consumption, and less potently inhibited gain of body weight, when food becomes incidentally available. Such feedback mechanisms operate more potently in low-altitude deer mice, which have better access to adequate food resources throughout the year. Why this sensitivity to HFD-induced inflammation targets specifically the skin and is not seen in other tissues remains puzzling, especially in view of the fact that, in the liver of deer mice, HFD robustly induces changes in the expression profiles of genes associated with the immune system, although inflammation does not proceed in a manner that can be detected histologically (Zhang et al., 2021). Yet, it appears that it possesses evolutionary significance in modulating appetite, in a subset of animals only, which may be relevant to their differential nutritional needs in different environments.

It is conceivable that these mechanisms, integrating variations in the UPR with the propensity for body weight gain, are also applicable to human populations in which highly variable prevalence of obesity has been recorded. Furthermore, they advocate for the performance of genomic studies raising the testable hypothesis that UPR modifiers may function as modulators of the propensity for body weight gain and obesity.

## MATERIALS AND METHODS

### Animals

Deer mice, *P. maniculatus bairdii* (BW Stock), were closed-colony bred in captivity since 1948 and descended from 40 ancestors wild caught near Ann Arbor, MI, USA. Sonoran deer mice, *P. maniculatus sonoriensis* (SM2 Stock), were derived from ∼50 animals wild caught by Jack Hayes in 1995 near White Mountain Research Center, Bishop, CA, USA (Havighorst et al., 2017). In this study, we picked 42 deer mice (33 males and nine females) and fed them either a regular chow diet or a HFD (58 kcal% fat and sucrose, Research Diets D12331) for 14 or 28 weeks, starting at 3-4 months of age. Some animals in this study also participated in a study reported recently (Zhang et al., 2021). Blood was extracted from the orbital sinus of anesthetized deer mice with a heparinized capillary tube (Fisherbrand). For serum extraction, the whole blood was placed in a blood collection tube (BD Microtainer) and centrifuged at 9500 ***g*** for 5 min. Serum samples were then aliquoted and stored at −80°C until the analyses. The animals were sacrificed using isoflurane as an anesthetic followed by cervical dislocation, and the brain, liver, skin and adipose tissue were collected. Tissues for RNA isolation were frozen using dry ice, and tissues for histology were fixed in 4% formaldehyde. All animal procedures complied with relevant national and institutional animal welfare laws, guidelines and policies, and were approved by the Institutional Animal Care and Use Committee (IACUC) and the Department of Health and Human Services, Office of Laboratory Animal Welfare, University of South Carolina (Approval No. 2349-101211-041917).

### Cell culture

Fresh ear punches were collected during routine weaning and marking procedures at ∼3 weeks of age. After the ear punch tissue was bathed in 70% ethanol for 2 min, it was placed in RPMI 1640 medium (HyClone) supplemented with 10% fetal bovine serum (Gibco), 100 U/ml penicillin, 100 μg/ml streptomycin and 0.292 mg/ml L-glutamine (HyClone), as previously described (Zhang et al., 2019). Ear punches were minced and then treated with 2 mg/ml collagenase type I (Millipore) for 1 h. Undigested debris was removed once cells were visible. The disassociated cells were cultured in the same medium at 37°C and in 5% CO_2_. Cells were passed when adherent layers reached 90% confluence and for no more than seven passages. For tunicamycin treatment, cells were split into six-well plates, at 300,000 cells/well, and cultivated for 24 h. Then cells were treated with tunicamycin (5 μg/ml) for 5 h, immediately followed by RNA extraction.

### RNA isolation, cDNA synthesis and quantitative reverse transcription PCR (RT-qPCR)

RNA was isolated with a Qiagen RNeasy Plus Mini Kit according to the manufacturer's recommendations. RNA was eluted using 30 μl nuclease-free water. Complementary DNA (cDNA) synthesis was performed using an iScript cDNA synthesis kit (Bio-Rad) according to the supplied protocol. RT-qPCR was performed on a T100 thermocycler (Bio-Rad) using iTaq Universal SYBR Green Supermix (Bio-Rad). Specific oligonucleotide primers for target gene sequences are listed in Table 1. Arbitrary units of target mRNA were normalized to the expression of *Gapdh*.

**Table 1.**
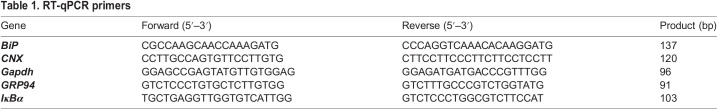
RT-qPCR primers

### ThT staining

Primary fibroblasts were split into six-well plates, at 300,000 cells/well, and cultivated for 24 h. Then, cells were treated with ThT (4 μM) and tunicamycin (5 μg/ml) for 5 h. ThT is known to be able to detect ER stress by exhibiting fluorescence when it binds to protein aggregates (Beriault and Werstuck, 2013). After washing and detaching with trypsin, the cells were collected with cold PBS. ThT fluorescence intensities were assessed using an LSRII flow cytometer (BD Biosciences).

### Histology

At sacrifice, the brain, liver, skin and adipose tissue were excised from each animal, fixed for 3-5 days in 4% formaldehyde, dehydrated with 100% ethanol and embedded in wax. Paraffin sections were stained with Hematoxylin and Eosin (H&E). Histological examination of the specimens was performed blindly by a certified pathologist. Images shown were obtained by an ICC50 HD (Leica Microsystems).

### Cytokine array

The serum samples of deer mice were pooled for each group, and the relative levels of serum cytokines and chemokines were assessed with a Mouse Cytokine Antibody Array C3 (RayBio C-Series). Blocking, biotinylated antibody cocktail and HRP-streptavidin incubation, washing conditions and chemiluminescent detection were performed according to the manufacturer's instructions.

### Leptin enzyme-linked immunosorbent assay (ELISA) assay

Blood samples of deer mice for leptin level detection were collected before HFD consumption and at 4 and 28 weeks after HFD administration. The concentration of serum leptin for each animal was measured using an Ultra Sensitive Mouse Insulin ELISA Kit (Crystal Chem) according to the manufacturer's instructions.

### Immunostaining

Paraffin-embedded skin tissues underwent antigen retrieval in 0.01 M citrate buffer (pH 6.0), permeabilization with 0.1% Triton X-100 and blocking with 10% goat serum. The sections were then incubated in phosphate-buffered saline with 0.1% Tween 20 and 1% bovine serum albumin at 4°C overnight with anti-vimentin (ab92547, Abcam; 1:250 dilution). After washing, the sections were incubated with goat anti-rabbit IgG H&L Alexa Fluor 488 (ab150077, Abcam; 1:1000 dilution) for 1 h at 25°C and then incubated with anti-BiP (ab21685, Abcam; 1:250 dilution) conjugated to Alexa Fluor 647 (ab269823, Abcam) at 4°C overnight. Images were obtained with a confocal microscope (Carl Zeiss LSM 700) and analyzed with ImageJ.

### Fatty acid treatment

Palmitic acid (PA; P0500, Sigma-Aldrich) and oleic acid (OA; O1008, Sigma-Aldrich) were dissolved in 100% ethanol and conjugated to 10% fatty acid-free bovine serum albumin (BSA) (A0281, Sigma-Aldrich) in RPMI 1640 medium (HyClone). The molar ratio of PA/OA:BSA is 6:1. The primary fibroblasts were treated with PA or OA at a concentration of 500 μM for 24 h.

### RNA sequencing

RNA and library preparation, sequencing, postprocessing of the raw data and data analysis were conducted by the University of South Carolina (USC) COBRE Center for Targeted Therapeutics Functional Genomics Core. RNAs were isolated with a Qiagen RNeasy Plus Mini Kit. RNA integrity was evaluated using an Agilent Bioanalyzer, and samples had a quality score ≥8.6. RNA libraries were prepared using established protocol with Next Ultra II Directional RNA Library Prep Kit for Illumina (NEB, Lynn, MA, USA). Each library was made with one of the TruSeq barcode index sequences and pooled together into one sample to be sequenced on the HiSeq 2×150 bp pair-ended sequencing platform (Genewiz). Sequences were aligned to the *P. maniculatus* genome [HU_Pman_2.1 (GCA_003704035.1)] in ensembl.org using STAR v2.7.2 (Dobin et al., 2013). Reads were counted using the featureCounts function of the Subreads package (Liao et al., 2013) using Ensembl GTF and summarized at exon, transcript or gene level. Only reads that were mapped uniquely to the genome were used. The results have been deposited in Gene Expression Omnibus (GEO; GSE179058). The flowchart of the transcriptome coordination analyses was described previously (Zhang et al., 2019; Soltanmohammadi et al., 2021). Briefly, the Pearson's correlation coefficients were calculated between the whole transcriptome and the transcripts indicated. Subsequently, the coordination between the selected pairs of transcripts was calculated as the correlation of their Pearson's R values and plotted as dot plot charts.

### Cholesterol assay

The levels of total, free, HDL and LDL/VLDL cholesterol in sera of HR and LR deer mice after 14 weeks of HFD administration were measured with a cholesterol assay kit (ab65390, Abcam) according to the manufacturer's instructions. The cholesterol concentrations were detected on a microplate reader at an optical density of 570 nm.

### Statistical analysis

Data were expressed as mean±s.e.m, unless specified otherwise. Results were analyzed using paired or unpaired two-tailed Student's *t*-test, one-way ANOVA with Tukey's multiple comparisons test or Pearson's correlation as indicated. Statistical analyses were conducted using Prism software (version 9.2.0; GraphPad Software). *P*<0.05 was considered statistically significant.

## Supplementary Material

Supplementary information
