# Supplementary material for: Propensity to endoplasmic reticulum stress in deer mouse fibroblasts predicts skin inflammation and body weight gain
Source: Dis Model Mech. 2021 Oct 18;14(10):dmm049113. doi: 10.1242/dmm.049113 (PMC8543066; doi:10.1242/dmm.049113)
Supplement: Supplementary information [file dmm-14-049113-s1.pdf]

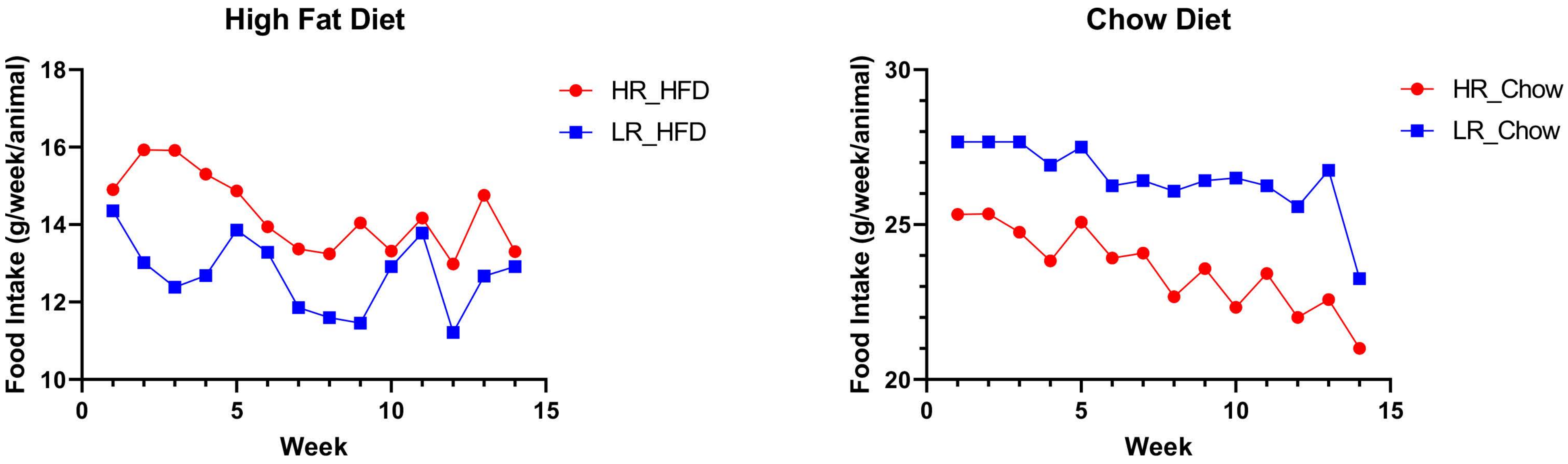

Fig. S1. High-fat diet or regular chow diet intake in HR (n=10, males) or LR (n=9, males) deer mice. The food consumption measured every week for each group of animals as they are required to be housed together.

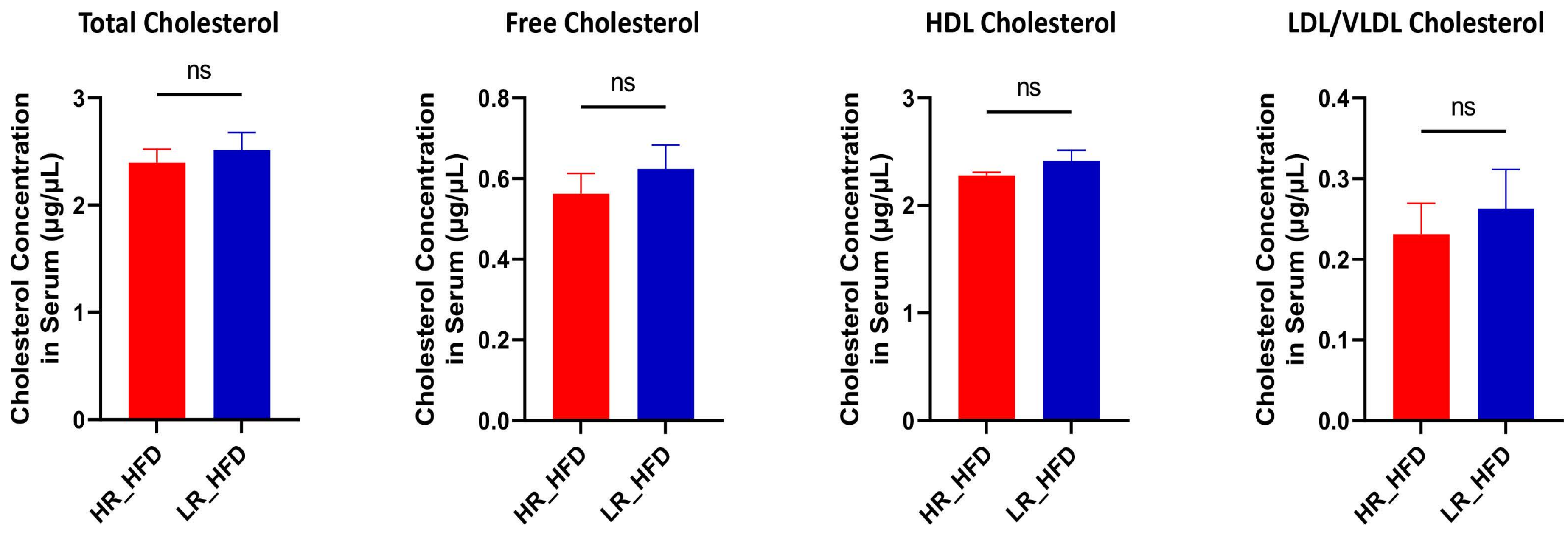

Fig. S2. Cholesterol levels in sera of deer mice fed with the HFD. The total, free, HDL and LDL/VLDL cholesterol concentrations in high (n=5, males) and low (n=5, males) responders of deer mice after 14 weeks of the HFD consumption measured by a cholesterol assay kit. The P values were calculated with unpaired t-test and none of them was statistically significant.

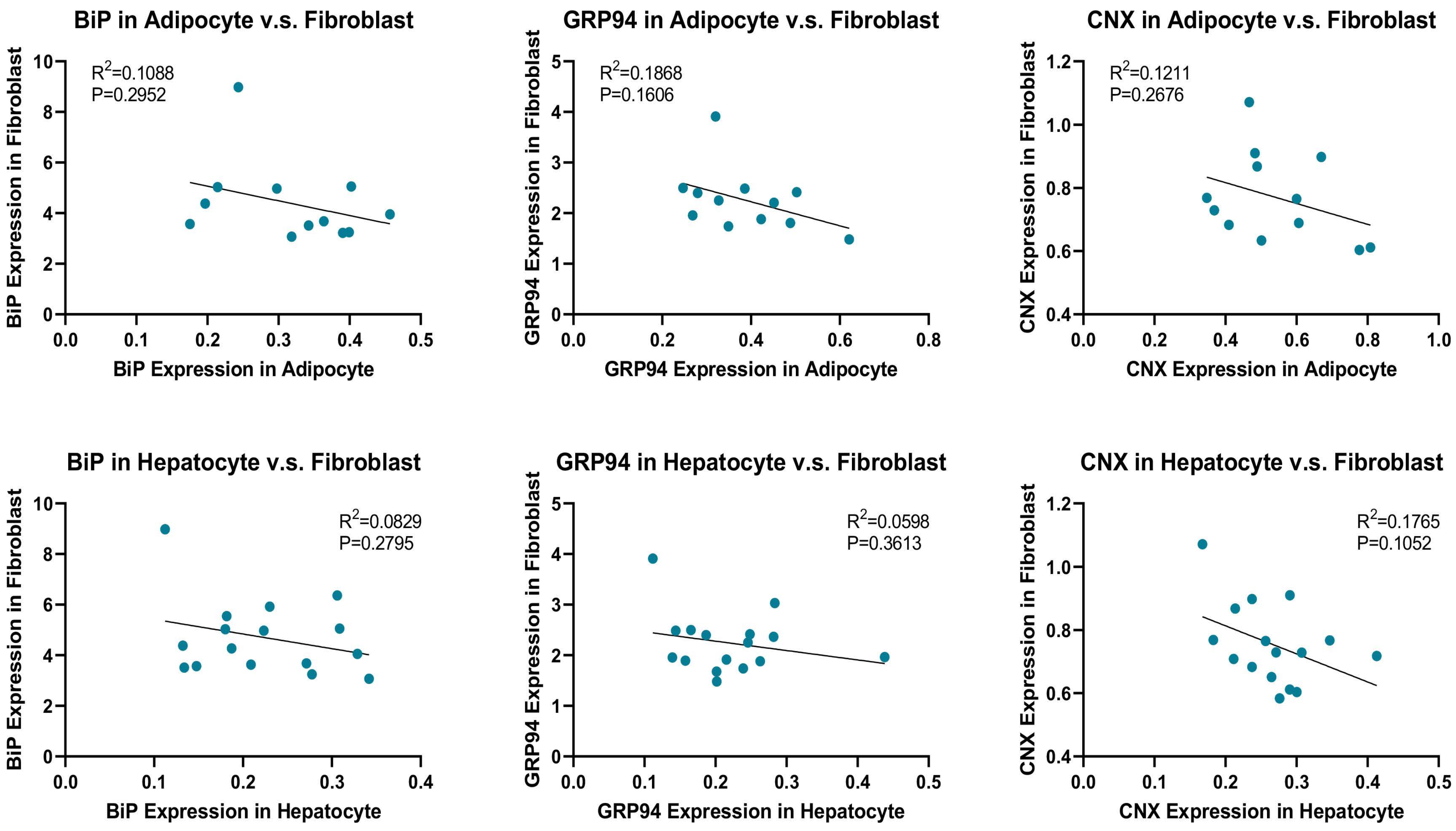

Fig. S3. Chaperone expression in cultured fibroblasts and peripheral tissues. Correlation of ER chaperones (BiP, GRP94 and CNX) expression between primary fibroblasts treated with tunicamycin and adipocytes/hepatocytes of deer mice after the HFD consumption measured by RT-qPCR. R square values from Pearson's correlation and P-values are shown.
